# Melatonin modulates the gene expression of WEE1 kinase and clock genes: a crosstalk between the molecular clocks of the placenta?

**DOI:** 10.3389/fendo.2025.1640635

**Published:** 2025-10-29

**Authors:** Carlos Venegas, Kevins Jara-Medina, Nicole Cueto, Gerardo Cabello-Guzmán, Constanza Lagunas, Luis Lillo, Francisco J. Valenzuela-Melgarejo

**Affiliations:** ^1^ Laboratory of Molecular Cell Biology, Department of Basic Sciences, Universidad del Bío-Bío, Chillán, Chile; ^2^ Department of Basic Sciences, Universidad del Bío-Bío, Chillán, Chile

**Keywords:** melatonin, placenta, clock gene, cell cycle, pregnancy

## Abstract

**Background:**

The circadian system organizes during 24 hours the temporal variations in biological processes such as the cell cycle, metabolism, and hormone production. This occurs by a transcriptional/translational feedback loop of core clock genes, namely, *BMAL1*, *PER1-3*, and *CRY1-2*. The CLOCK–BMAL1 complex regulates clock-controlled genes like WEE1 kinase, a key modulator of mitotic entry and placental cell proliferation.

**Objective:**

We aimed to identify temporally regulated gene expression patterns in the human placenta using bioinformatics analysis of available microarrays in Gene Expression Omnibus (GEO) datasets and to validate selected findings in cultured placental explants.

**Methods:**

Temporal microarray data from the GEO were analyzed to identify circadian and cell cycle-related genes. Selected targets were validated *in vitro* using explant cultures of human placenta sampled every 4 hours for 36 hours, with or without 10 nM melatonin.

**Results:**

We observed rhythmic expression of *BMAL1*, *PER1*, *PER2*, and *WEE1* in human placental explants, consistent with the temporal patterns detected *in silico*. Melatonin treatment suppressed the circadian oscillation of *BMAL1*, *PER2*, and *WEE1*. Interestingly, the placenta produced melatonin steadily over 36 hours, and exogenous melatonin did not alter this production.

## 1 Introduction

The coordinated function of the circadian system and the cell cycle is critical for cell development, homeostasis (1–3), and tissue regeneration (1, 4–7). The disruption of circadian rhythms elevates the cancer risk (8) due to the impaired expression of the target genes from the cell cycle, i.e., cyclins, proto-oncogenes, and tumor suppressor genes (7, 9–15).

The central clock of the circadian system resides in the suprachiasmatic nucleus (SCN) (16–21), which synchronizes the peripheral oscillators through neural and humoral pathways. The primary humoral signal used by the body is the pineal hormone melatonin, a hormone synthesized during dark hours and playing a central role as a systemic timekeeping (22–30).

At the molecular level, circadian oscillations depend on a transcriptional/translational feedback loop involving a group of clock genes, namely, *BMAL1* (also known as *ARNTL*), *CLOCK*, *PER1-3*, and *CRY1-2* (31, 32). The CLOCK/BMAL1 heterodimeric complex initiates the circadian transcription by binding to conserved promoter sequences, namely, E-box (CACGTG) from clock genes *PER1–3* and *CRY1-2* (31), thereby giving the circadian output signals to clock-controlled genes. One such target is the kinase WEE1, which can inhibit Cdc2-cyclin B complexes, delaying G2/M transition and modulating cell proliferation in a time-dependent manner (1, 7).

Like the circadian system, the cell cycle is a finely timed and temporal process capable of generating a coordinated series of cell divisions, regulated by cyclin-dependent protein kinases (Cdk) essential for the stage transition (33–35). *WEE1* is of particular interest because its promoter contains E-box motifs responsive to CLOCK/BMAL1, positioning it at the interface between circadian clock and cell cycle regulation (1, 7, 36–39).

The placenta is an endocrine tissue with a circadian production of hormones essential for pregnancy maintenance (40–43). The disruption of these temporal events has been linked to altered trophoblast proliferation, differentiation, and invasion (44, 45). All those temporal events are hallmarks of placental pathologies during pregnancy (31) and placental tumors (46–49).

Studies in trophoblast cells, previously stimulated by serum shock, have shown the circadian expression of the clock gene *PER2* (50, 51) and the *ex vivo* expression of *CLOCK*, *BMAL1*, and *PER1-2* (52–55). Moreover, maternal melatonin can cross the placental barrier, exhibiting a diurnal rhythm during pregnancy, suggesting that it can give a chronobiotic signal to the placenta (31, 56–60). Interestingly, shift work modifies the temporal production of melatonin, which increases cancer incidence, suggesting an association between melatonin secretion, oncogenesis, and cell proliferation (61–66).

Recent transcriptomic datasets available in the Gene Expression Omnibus (GEO) provide the opportunity to explore temporal data of differentially expressed genes (DEGs) in the placenta, showing potential targets critical for placental physiology. DEG analysis can provide insight into the crosstalk between the circadian system and the cell cycle. We found common pathways modified by time hours and further investigated using placental culture and quantitative PCR. In this context, we speculated that the human placenta clocks can be modified by melatonin supplementation. These can determine the circadian output of critical signals to clock-controlled genes like the cell cycle regulator WEE1.

The placenta expresses melatonin-synthesizing enzymes such as AANAT and ASMT, as well as melatonin receptors, and maternal melatonin can cross the placental barrier (57, 67, 68). These findings provide a biological rationale for testing the effects of exogenous melatonin on placental circadian gene expression.

## 2 Materials and methods

### 2.1 Data source and bioinformatics analysis

The bioinformatics analysis was designed to identify time-dependent placental DEGs enriched for circadian and cell cycle pathways across independent GEO datasets. We analyzed datasets from the Gene Expression Omnibus (http://www.ncbi.nlm.nih.gov/geo) similar to what was reported previously (69, 70) for the terms “placenta and clock”, “placenta and circadian”, and “trophoblast and culture” (n = 139). We excluded platform data without temporal samples or incomplete incoming data. “GSE86171”, “GSE60433”, and “GSE40182” include temporal samples between 0 and 48 hours that were visualized using GEO Profiles graphics and the parameter Benjamin and Hochberg false discovery rate methodology with significance thresholds set at log2 fold change (logFC) ≥1 and adjusted p-value <0.05. We utilized the Kyoto Encyclopedia of Genes and Genomes (KEGG) for the functional analysis of cell cycle and circadian rhythms. In the present study, we collected, combined, and identified the gene expression profile using a Venn diagram. p < 0.05 was considered a significant difference by employing DAVID Bioinformatics 6.8, released Oct. 2016. The GO terms were “circadian rhythms”, “circadian regulation of gene expression”, “regulation of circadian rhythm”, “entrainment of the circadian clock by photoperiod”, and “cell cycle”.

### 2.2 Human placental tissue collection and culture

Term placentas from uncomplicated vaginal deliveries were obtained at approximately 07:00 hours at Herminda Martín Hospital (Chillán, Chile) after written informed consent was provided. The Ethics Committee approved the protocols of the Hospital and the University of the Bío Bío. Placentas were maintained at 4°C and processed at 07:00 hours. The tissue was washed three times with ice-cold phosphate-buffered saline (PBS) to eliminate red blood cells and trimmed to obtain a fetal portion of the placenta (chorion). Fifty-four explants of approximately 2 mm (L) × 2 mm (W) × 2 mm (H) and a mass of 45 ± 0.841 mg (wet mass) were used, according to the protocol of Cemerikic et al. (71).

Explants were cultured individually following previously described protocols (71–73). They were preincubated in M-199 medium (pH 7.2) and maintained in a humidified environment at 37°C and 5% CO_2_ for 4 hours. Then, they were transferred to fresh medium either alone (control) or supplemented with 10 nM melatonin (treatment group). The concentration of 10 nM melatonin was selected as a physiologically relevant dose, within the range used in previous studies on peripheral tissues (e.g., 10–100 nM) (74–79). This lower concentration was chosen to avoid potential pharmacological effects while maintaining biological activity. A sampling of three explants and the supernatant was conducted every 4 hours. All explants were weighed and stored with 1 mL TRIzol reagent (Invitrogen, Invitrogen Corporation, Carlsbad, California, USA). Explants and supernatant were stored frozen at −20°C.

### 2.3 Extraction of total RNA and reverse transcription (RT-PCR)

Explants of the human placenta were extracted in two stages: i) by the TRIzol method modified following the manufacturer’s instructions (80) (phase separation, precipitation, and washing RNA) to the ethanol phase and later and ii) extraction using Kit SV Total RNA Isolation System modified following the instructions of the manufacturer (Promega Corporation, Madison, Wisconsin, USA) (purification of RNA). The absorbance was measured at 260 and 280 nm using a spectrophotometer to determine the concentration of RNA. Reverse transcription of 20 ng of extracted RNA was performed using the Improm Kit II Reverse Transcription System (Promega, Promega Corporation, Madison, Wisconsin, USA) in a final volume of 20 µL. The reverse transcription was at 70 °C for 5 minutes, 4°C for 5 minutes, 25°C for 5 minutes, 42°C for 60 minutes, and 70°C for 15 minutes.

### 2.4 Quantitative real-time PCR

The relative expression of the mRNAs of clock genes *BMAL1*, *PER1-2*, and *WEE1* was measured in samples of total cDNA. The PCR was performed in a final volume of 10 µL containing 0.33 µL of primers, forward and reverse primers of the genes studied, 3.8 µL of nuclease-free H_2_O, and 5.5 µL of Master Mix II SYBR Brilliant Green (Agilent Technologies, Santa Clara, California, USA). The following primers were used: *BMAL1*, forward, 5′-CTGCATCCTAAAGATATTGCCAAAG-3′, and reverse, 5′-GTCGTGCTCCAGAACATAATCG-3′; *PER1*, forward, 5′-GGGCAAGGACTCAGAAAGAA-3′, and reverse, 5′-AGGCTCCATTGCTGGTAGAA-3′; *PER2*, forward, 5′-TGGATGAAAGGGCGGTCCCT-3′, and reverse, 5′-ACTGCAGGATCTTTTTGTGGA-3′; *WEE1*, forward, 5′-CGCGATGAGCTTCCTGAGCCG-3′, and reverse, 5′-CAGCGCACCGGCGAGAAAGAG-3′; cyclophilin, forward, CTCCTTTGAGCTGTTTGCAG-3′, and reverse, 5′-CACCACATGCTTGCCATCC-3′. For expression from quantitative real-time PCR (qPCR) data, all expression was normalized with cyclophilin for calculating relative gene expression by double delta Ct (ΔΔCt) and transformed to 2^−ΔΔCt^.

### 2.5 Melatonin measurement

The supernatant was cleaned with activated charcoal and measured by spectroscopic imaging using Fourier transform infrared (FTIR) spectroscopy associated with Attenuated Total Reflectance (ATR) (ATR–FTIR). Spectral measurements of the melatonin standard curve at 0.3–3,000 nM (Sigma-Aldrich, St. Louis, Missouri, USA.) were conducted, and supernatant samples were measured in triplicate using ATR–FTIR. The sample spectrum of 10 μL was recorded at room temperature in the region 1,000–4,000 cm^−1^ directly on a JASCO FT/IR-4100 Fourier transform infrared spectrophotometer with a 4.0 cm^−1^ resolution. A linear relationship was found for melatonin measurement at 1,492 cm^−1^. The melatonin content was calculated following the methodology described by Filali et al. (81). The inter-assay and intra‐assay coefficients of variation were less than 18%. Endogenous melatonin was quantified in explants maintained without supplementation. Exogenous melatonin levels were evaluated in explants supplemented with 10 nM melatonin. Paired untreated controls and the standard curve were used to differentiate between the hormone secreted by the tissue and the exogenous melatonin added to the medium.

### 2.6 Statistical data analysis

Data were expressed as mean ± SEM and analyzed using repeated-measures ANOVA, followed by Newman–Keuls *post-hoc* test, or Student’s t-test as appropriate. Rhythmicity in gene expression was evaluated using non-linear regression of the sine-wave function expressed as Y = Baseline + Amplitude * Sine (Frequency X + Phase shift). All data were normalized between 0 and 1; the data were analyzed using the GraphPad Prism 5 software, and p < 0.05 was considered statistically significant.

## 3 Results

### 3.1 Identification and functional classification of differentially expressed genes

To explore whether circadian and cell cycle pathways were consistently represented in placental gene expression, we first analyzed publicly available transcriptomic datasets (GEO). We first asked whether time of day-dependent transcriptional changes in placental tissue preferentially involve circadian and cell cycle pathways across independent datasets. The expression profiling dataset of mRNA (GEO database) gives the tools for bioinformatics analysis of molecular pathways modified by time hours in the placenta. We performed the identification of DEGs via GO term enrichment and functional classification using DAVID. We selected the GO classification related to the circadian system and cell cycle. We used terms such as “cell cycle”, “circadian rhythms”, “circadian regulation of gene expression”, “regulation of circadian rhythm”, and “entrainment of the circadian clock by photoperiod”. We detected three complete microarray experiments for analysis: “GSE86171”, “GSE60433”, and “GSE40182”. The periods studied in the microarrays were 0, 3, 12, 24, and 48 hours.

**Figure 1:**
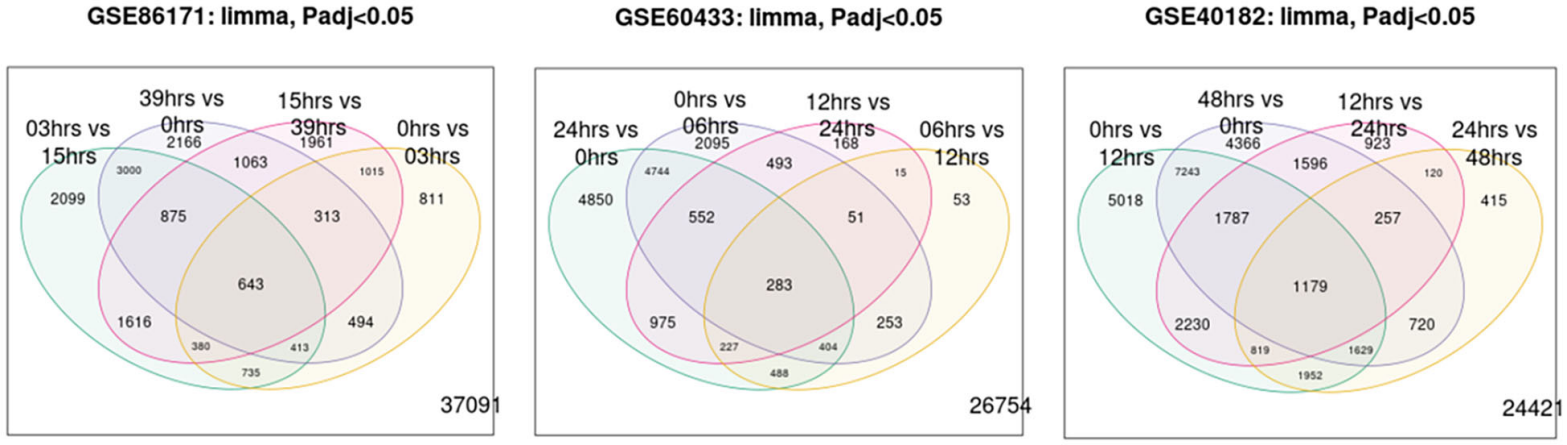
A Venn diagram of genes between Gene Expression Omnibus (GEO) and the time of day.

**Figure 2:**
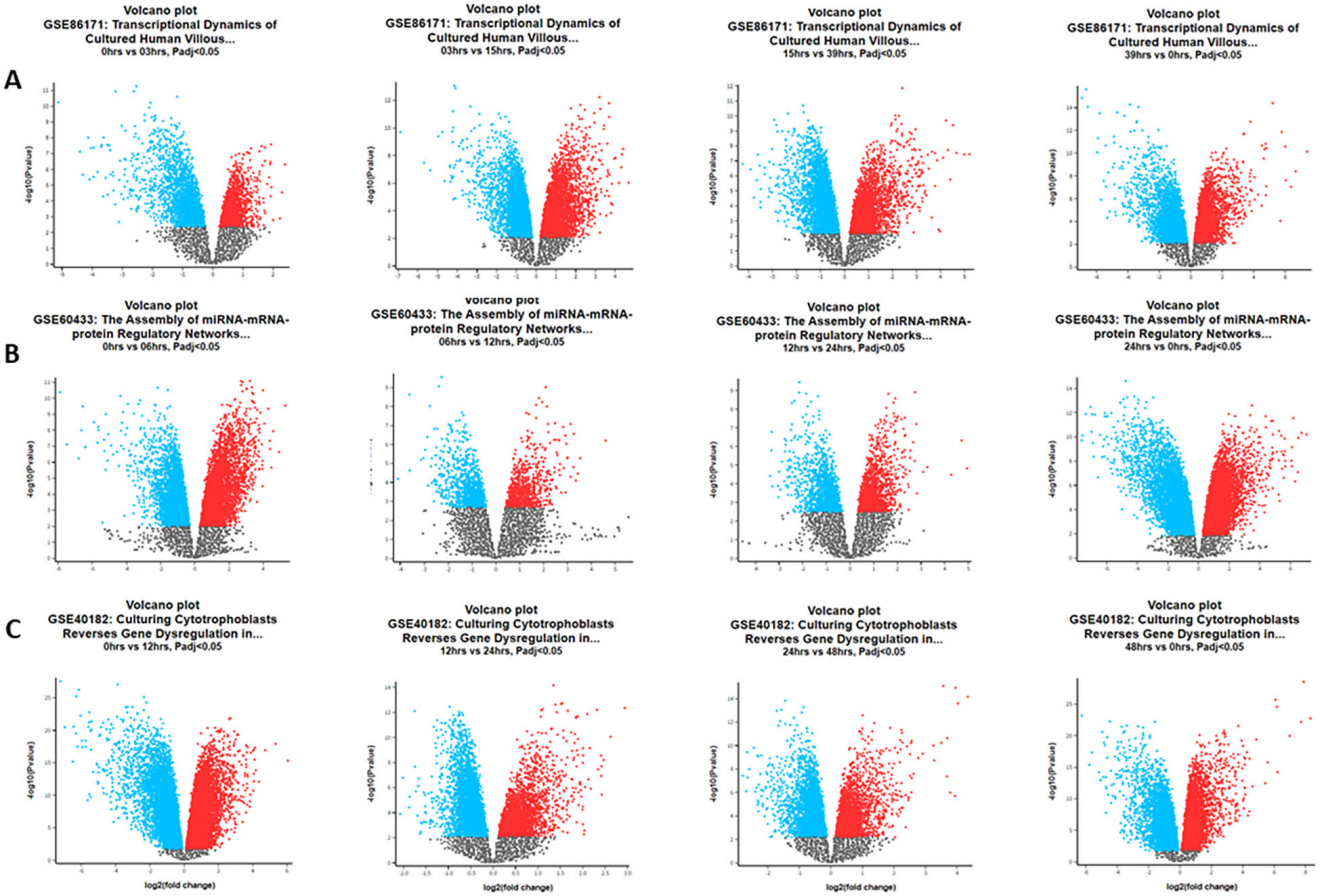
Volcano plots of differentially expressed genes in the Gene Expression Omnibus (GEO) datasets during the hour of the day. **(A)** Data GSE86171: at 0 vs. 3 hours, we detected 2,381 upregulated and 2,418 downregulated genes. At 3 vs. 15 hours, we detected 4,583 upregulated and 5,162 downregulated genes. At 15 vs. 39 hours, we detected 3,385 upregulated and 4,474 downregulated genes. At 39 vs. 0 hours, we detected 4,701 upregulated and 4,250 downregulated genes. **(B)** Data GSE60433: at 0 vs. 6 hours, we detected 4,523 upregulated and 4,344 downregulated genes. At 6 vs. 12 hours, we detected 824 upregulated and 949 downregulated genes. At 12 vs. 24 hours, we detected 1,407 upregulated and 1,356 downregulated genes. At 24 vs. 0 hours, we detected 6,286 upregulated and 6,224 downregulated genes. **(C)** Data GSE40182: at 0 vs. 12 hours, we detected 10,932 upregulated and 10,019 downregulated genes. At 12 vs. 24 hours, we detected 3,426 upregulated and 5,483 downregulated genes. At 24 vs. 48 hours, we detected 3,215 upregulated and 3,875 downregulated genes. At 48 vs. 0 hours, we detected 10,017 upregulated and 8,755 downregulated genes.

Functional annotation using DAVID identified 391 common DEGs (60.9%) during all time hours studied. Functional enrichment analysis (DAVID/KEGG) of the 391 common DEGs revealed significant overrepresentation of the “circadian rhythm” and “cell cycle” pathways (adjusted p < 0.05). Additional enriched terms included apoptosis and DNA repair, consistent with the central role of circadian regulation in cell proliferation and survival. **Figure 1** shows the Venn diagram demonstrating the intersections of genes at different times of the day. Approximately 643, 283, and 1,179 common genes changed their expression level over all the time hours studied. Volcano plots for each dataset (**Figures 2A–C**) display the distribution of DEGs over time, and the pattern is visualized at every time studied in “GSE86171”, “GSE60433”, and “GSE40182”. Similarly, the data of clock genes and regulators of the cell cycle for log2(fold change) and −log10(p-value) are shown in **Table 1** for every time hour. The relative expression values suggest the time variation of clock gene expression in the placenta for at least 24 hours, with a peak for *PER2* and *CRY1* during the first half of the day. *BMAL1* shows a peak early in the morning, and the cell cycle genes *TP53*, *CIPC*, and *WEE1* show a peak during the interval between early in the morning and noon, suggesting a temporal variation of genes of circadian and cell cycle clocks.

**Table 1:** Volcano data of differentially expressed genes in the circadian system and cell cycle GEO datasets.

GEO dataset	Gene	0–3 hours log2(fold change) and −log10(p-value)	3–15 hours log2(fold change) and −log10(p-value	15–39 hours log2(fold change) and −log10(p-value)	39–0 hours log2(fold change) and −log10(p-value)
GSE86171	*Per1*	n.d	n.d	n.d	n.d
	*Per2*	−0.492	2.623	0.767*	3.558*
	*Per3*	n.d	n.d	n.d	n.d
	*BMAL1*	−0.88	2.479	0.990*	2.884*
	*BMAL2*	n.d	n.d	−1.794*	6.259*
	*Clock*	n.d	n.d	n.d	n.d
	*Cry1*	−1.942	9.112	1.673	8.208
	*Cry2*	n.d	n.d	n.d	n.d
	*TP53*	0.498*	2.848*	−1.099	6.48*
	*CIPC*	n.d	n.d	0.95	5.198
	*WEE1*	n.d	n.d	n.d	n.d
GSE60433	*Per1*	3.12	6.69	n.d	n.d
	*Per2*	3.114*	6.844*	−2.719*	6.002*
	*Per3*	1.957	4.331	−1.868	3.939
	*BMAL1*	1.468	5.996	1.226	4.895
	*BMAL2*	n.d	n.d	n.d	n.d
	*Clock*	n.d	n.d	n.d	n.d
	*Cry1*	2.433	9.05	n.d	n.d
	*Cry2*	1.602	6.211	−1.115	4.262
	*TP53*	n.d	n.d	n.d	n.d
	*CIPC*	n.d	n.d	n.d	n.d
	*WEE1*	n.d	n.d	n.d	n.d
GSE40182	Per1	n.d	n.d	n.d	n.d
	Per2	0.219	1.833	n.d	n.d
	Per3	n.d	n.d	n.d	n.d
	BMAL1	n.d	n.d	n.d	n.d
	BMAL2	−2.048*	16.226*	n.d	n.d
	Clock	0.408*	4.975*	−0.403*	3.20*
	Cry1	n.d	n.d	n.d	n.d
	Cry2	n.d	n.d	n.d	n.d
	TP53	−0.336	2.587	n.d	n.d
	CIPC	0.938	7.955	n.d	n.d
	WEE1	0.552	1.95	n.d	n.d

We used four timepoints for data analysis. Clock- and cell cycle-related DEGs selected from the set of 391 time-regulated genes identified across GEO datasets. Values show log2(fold change) and −log10(p-value) at the indicated timepoint contrasts. These genes were prioritized to illustrate circadian–cell cycle crosstalk within the broader DEG set.

n.d, not detected; GEO, Gene Expression Omnibus; DEGs, differentially expressed genes.

(*) Mean of several data.

### 3.2 *In vitro* expression of clock gene and the WEE1 gene in human placental explants

**Figure 3:**
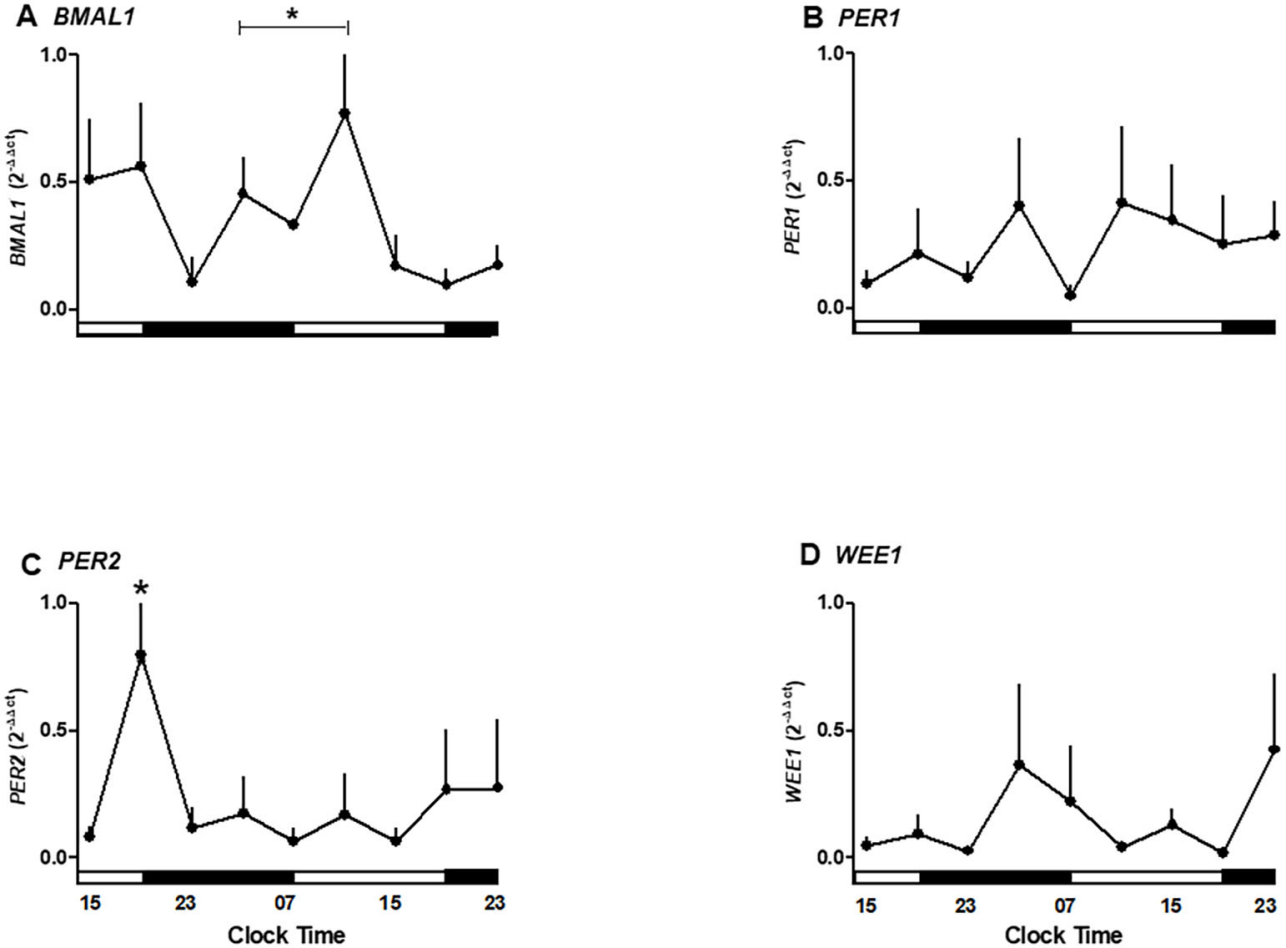
Temporal expression of clock and cell cycle genes in human placental explants cultured for 36 hours. **(A)** BMAL1 mRNA expression showing significant variation between 03:00–11:00 h and 15:00–23:00 h. **(B)** PER1 mRNA expression showing no significant oscillation but a trend toward higher levels in the evening. **(C)** PER2 mRNA expression peaking at 19:00 h during the first day of culture (p < 0.05). **(D)** WEE1 mRNA expression showing a mild, non-significant increase during nighttime hours.Data are expressed as mean ± SEM (n = 3 per timepoint). Statistical analysis by one-way ANOVA followed by Newman–Keuls post hoc test; p < 0.05 considered significant.

We next examined whether placental explants maintained circadian oscillations of core clock genes and the cell cycle regulator *WEE1* in culture. To validate the *in silico* observations associated with temporal variations observed in the microarray of the placenta, we cultured human placental explants and measured gene expression every 4 hours for 36 hours. We observed that the *BMAL1*, *PER1*, *PER2*, and *WEE1* genes maintain their mRNA expression in the culture of the human placenta for at least 36 hours (**Figure 3**).

As shown in **Figure 3A**, clock gene *BMAL1* expression increases during daylight hours, showing a rise between 03:00 and 11:00 hours. Also, *BMAL1* showed a local peak at 11:00 hours (range 03:00–11:00 hours is different from 15:00–23:00 hours of the second day of culture; p < 0.05, one-way ANOVA and Newman–Keuls post-test), whereas this expression showed a local minimum at 23:00 hours. The relative mRNA expression of *PER1* showed no significant changes during the hours studied but exhibited a trend toward higher expression in the evening (**Figure 3B**).

*PER2* expression changed during the hours of culture, showing a peak expression at 19:00 hours on the first day (p < 0.05 ANOVA and Newman–Keuls) and low expression levels in the following hours studied (**Figure 3C**). Wee1 expression showed no significant differences but trended upward during nighttime hours (**Figure 3D**).

The temporal data suggest an endogenous oscillation in *BMAL1* and *PER2* occurring in antiphase with a ~12-hour interval, indicative of a functional circadian clock in placental tissue.

### 3.3 Melatonin inhibits the expression of clock genes and the WEE1 gene

**Figure 4:**
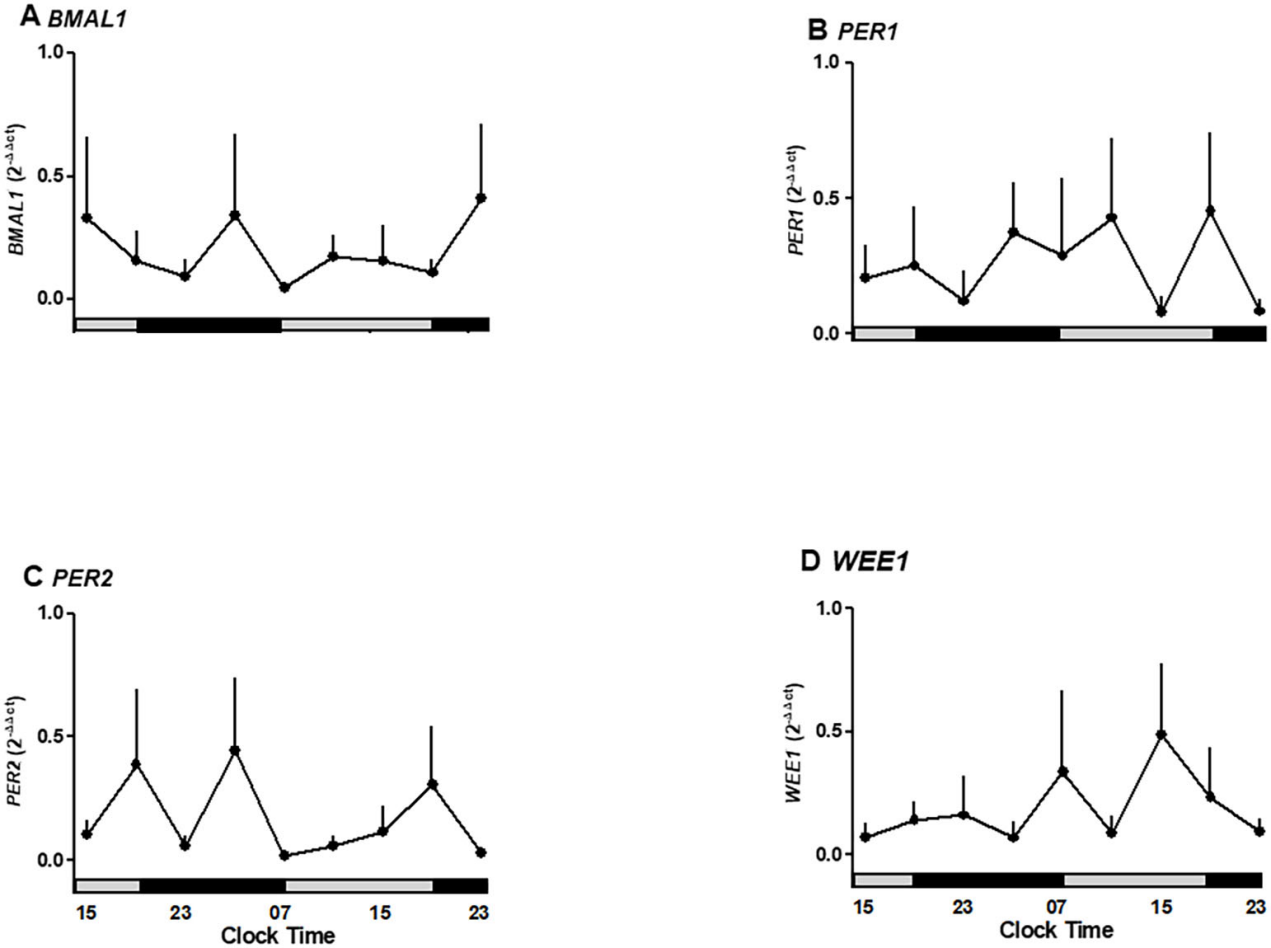
Oscillatory expression of clock genes BMAL1, PER1-2, and WEE1, a cell cycle gene in human placenta explants cultured for 36 hours in medium alone or plus melatonin. **(A)** BMAL1 expression under control and melatonin showing suppression of rhythmic peaks. **(B)** PER1 expression unaffected by melatonin treatment. **(C)** PER2 expression showing inhibition of oscillatory peaks by melatonin. **(D)** WEE1 expression showing a non-significant reduction under melatonin treatment.Profiles are representative of three placentas and expressed as Mean ± SE from 2-ΔΔCt. The bars on the X-axis indicate the relative hours of light (gray) and the hours of darkness (black).

Given that the placenta expresses the capacity to synthesize melatonin and receptors, we tested whether exogenous melatonin modulates the oscillations of *BMAL1*, *PER2*, and *WEE1*. The exposure of placental explants to 10 nM melatonin suppressed the rhythmic peaks of *BMAL1* and *PER2* expression observed in untreated cultures. Although *PER1* and *WEE1* did not show statistically significant changes, *BMAL1* expression was reduced between 07:00 and 19:00 hours under melatonin treatment (**Figures 4A–D**). These results suggest that exogenous melatonin can reduce the amplitude of circadian gene expression in placental tissue.

### 3.4 Oscillatory ratios reveal phase relationships between clock genes

**Figure 5:**
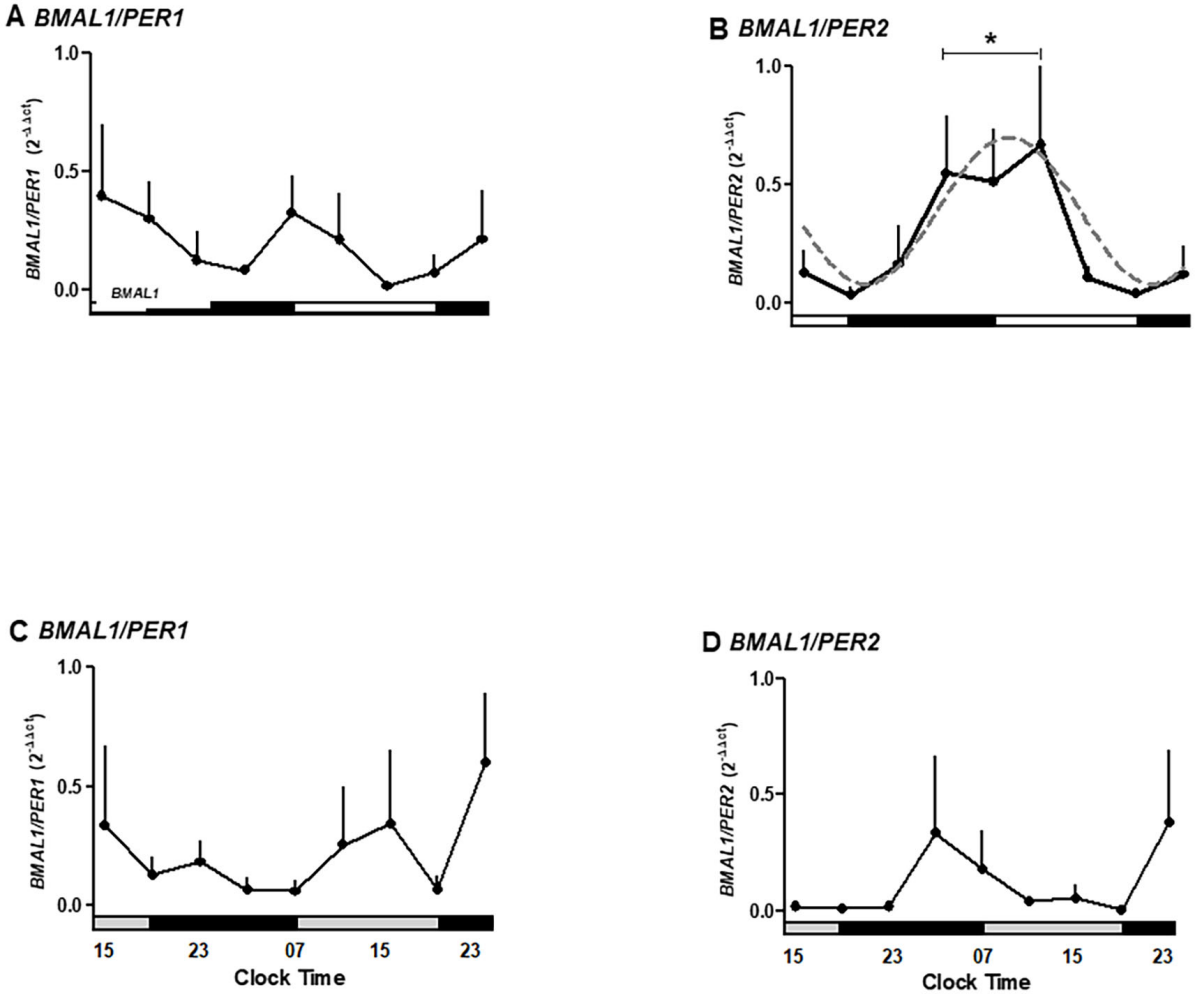
The ratio for expression of clock genes *BMAL1*, *PER1-2*, and *WEE1*, a cell cycle gene, in human placental explants cultured for 36 hours in medium alone **(A, B)** or medium plus melatonin **(C, D)**. Profiles are representative of three placentas and expressed as mean ± SE from 2^−ΔΔCt^. The dashed line in panel B represents the theoretical sine-wave function determined by equation Y = Baseline + Amplitude * Sine (Frequency X + Phaseshift), where Baseline = 0.39, Amplitude = 0.3, Frequency = 0.26, and Phaseshift = −0.8 for *BMAL1/PER-2* (r^2^ = 0.7368). The data were normalized, considering the highest individual value within the experiment as 1 and the lowest value as 0. The bars on the X-axis indicate the hours of light (white), the hours of darkness (black), and relative hours of light (gray, **A, B)**. * Different from other hours, one-way ANOVA, n = 3.

To further capture phase relationships among clock genes, we calculated BMAL1/PER1 and BMAL1/PER2 ratios across timepoints. To further evaluate gene oscillations, we calculated the expression ratios *BMAL1/PER1* and *BMAL1/PER2* (**Figure 5**). The circadian oscillation circuits are dependent on the transcriptional/translational feedback loop of clock genes, which act as positive and negative regulators, inducing/inhibiting their expression. *BMAL1/PER1* ratios showed non-significant variation but tended to peak at 15:00 and 23:00 hours on the second day of incubation (**Figure 5A**).

Moreover, *BMAL1/PER2* ratios exhibited significant oscillation, peaking between 03:00 and 11:00 hours and declining between 15:00 and 23:00 hours (p < 0.05; **Figure 5B**), fitting a sine-wave function (r^2^ = 0.7368). In contrast, melatonin treatment inhibited the *BMAL1/PER2* peaks (**Figures 5C, D**).

These findings support the existence of an antiphase rhythm between *BMAL1* and *PER2*, a circadian pattern that is disrupted by melatonin.

### 3.5 BMAL1/WEE1 ratio suggests a circadian regulation of the cell cycle

**Figure 6:**
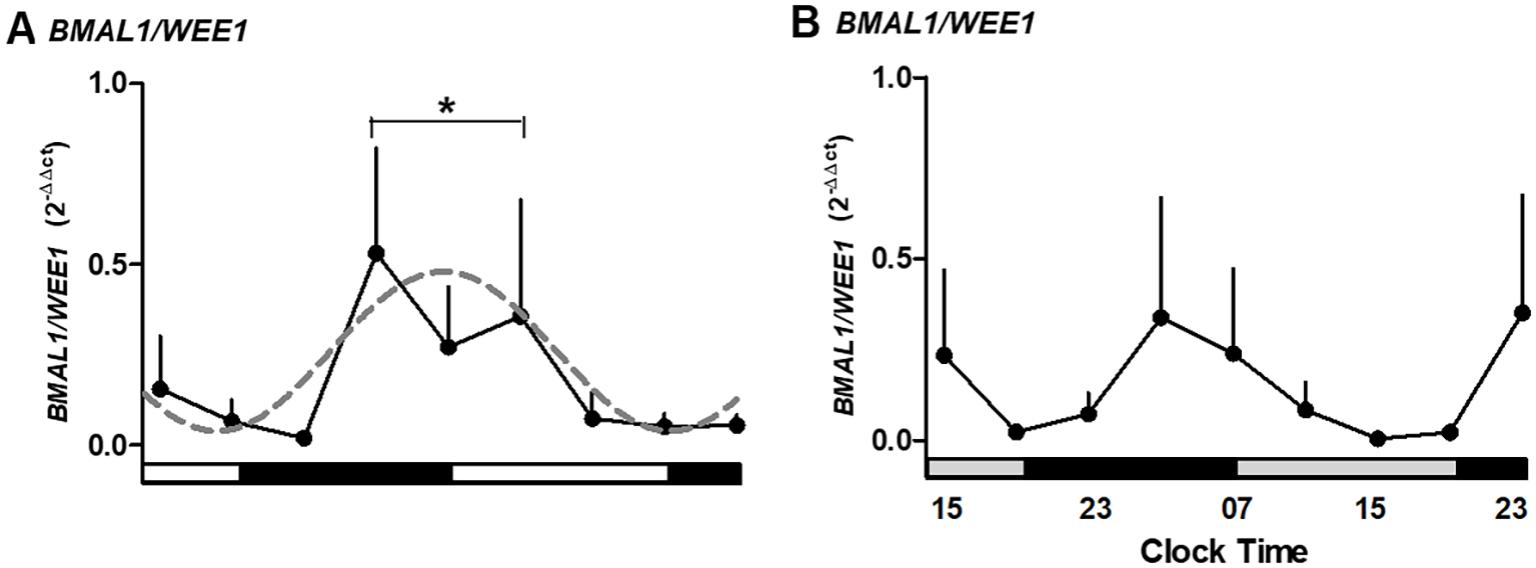
The ratio for expression of *BMAL1/WEE1* in medium alone **(A)** or medium alone plus melatonin **(B)** in human placental explants cultured for 36 hours. The dashed line in panel A represents the theoretical sine-wave function determined by equation Y = Baseline + Amplitude * Sine (Frequency X + Phaseshift), where Baseline = 0.26, Amplitude = 0.22, Frequency = 0.25, and Phaseshift = −0.08 for *BMAL1/WEE1* (r^2^ = 0.5304). Profiles are representative of three placentas and expressed as mean ± SE from 2^−ΔΔCt^. The data were normalized, considering the highest individual value within the experiment as 1 and the lowest value as 0. The bars on the X-axis indicate the hours of light (white), the hours of darkness (black), and the relative hours of light (gray, in panel B). * Different from other hours, one-way ANOVA, n = 3.

To assess circadian gating of the cell cycle, we analyzed the ratio of *BMAL1* to *WEE1* expression across the culture period. The *BMAL1/WEE1* expression ratio revealed a peak at 03:00–11:00 hours, followed by a decline during the night hours of the second day of culture (p < 0.05; **Figure 6A**). This antiphase relationship between *BMAL1* and *WEE1* was lost in melatonin-treated explants (**Figure 6B**). The pattern is consistent with the transcriptional regulation of *WEE1* by the CLOCK/BMAL1 complex.

### 3.6 Sustained melatonin production in placental explants

**Figure 7:**
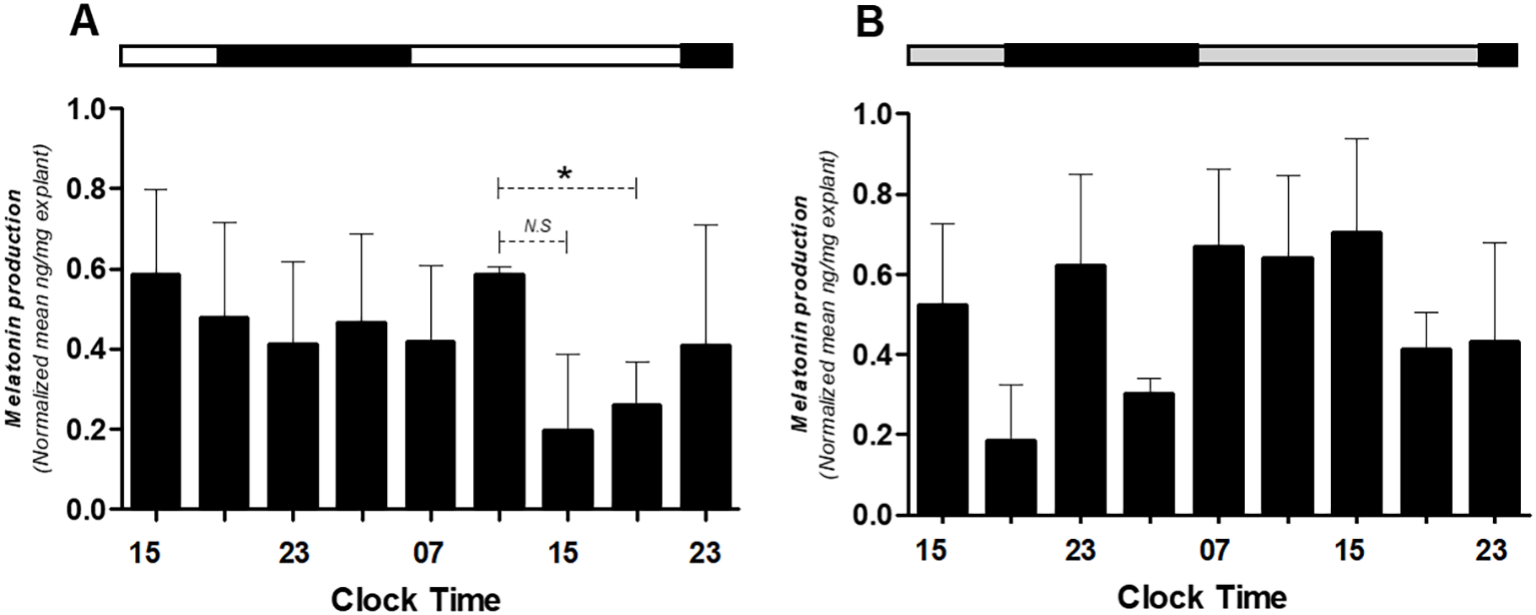
Melatonin production in medium alone **(A)** or medium alone plus melatonin **(B)** in human placental explants cultured for 36 hours. The average of each bar represents the average measurement of the melatonin concentrations obtained from the supernatant of each explant. The bar at the upper side of the graph indicates the hours of light (white), the hours of darkness (black), and relative hours of light (gray, in panel B). * Different from other hours, t-test, n = 3. N.S., non-significant difference.

Finally, it was assessed whether placental explants produce melatonin endogenously and whether supplementation alters secretion levels. Endogenous melatonin was quantified in supernatants from untreated cultures (**Figure 7A**), while apparent exogenous levels were assessed in melatonin-supplemented cultures (**Figure 7B**). Values in treated conditions were interpreted relative to the standard curve and to paired untreated controls to distinguish secretion from supplemented hormone.

These findings confirm that the human placenta can produce melatonin autonomously and suggest a regulatory feedback loop between melatonin and the placental clock.

## 4 Discussion

The placenta exhibits a circadian production of critical hormones required for a healthy pregnancy (41–43, 68, 82, 83). In agreement with these findings, Diallo et al. (68) demonstrated that the human placenta displays circadian oscillations in its metabolism and is capable of synthesizing the melatonin hormone, suggesting the presence of a functional circadian clock in the placenta. Importantly, impaired circadian rhythms in the placental physiology due to the inhibition of melatonin production (e.g., shiftwork and night-time light exposure) have been associated with pregnancy complications and adverse outcomes (31, 84–89). We selected a low nanomolar concentration (10 nM) of melatonin, consistent with prior studies in several tissues, where nanomolar doses are biologically active while avoiding potential pharmacological effects (74–79). Using bioinformatics analyses, we detected time-dependent variation in the expression of cell cycle genes *TP53*, *CIPC*, and *WEE1* and clock genes *PER2*, *PER3*, *CRY1*, and *BMAL1*. These results suggest that such genes may serve as markers to study the intrinsic oscillatory capacity of placental tissue *in vitro*. To further explore this capacity, we performed culture experiments with human placental explants. Also, we provided complementary *in silico* and *ex vivo* evidence that human placental tissue displays intrinsic circadian dynamics involving *BMAL1*, *PER2*, and *WEE1* and that melatonin reduces the amplitude of these rhythms.

In culture, explants of the placenta can maintain the cellular function between 24 and 72 hours, synthesizing critical factors such as human placental lactogen (72, 90), human chorionic gonadotropin (CG) (71), prorenin (91), angiogenin (92), placental 24,25(OH)_2_D_3_ (93), and NO (94), and also showing the capacity of l-tryptophan transport and indoleamine 2,3-dioxygenase activity (95). Similarly, in our explants of the human placenta, we detected the expression of clock genes *BMAL1* and *PER1-2*. We observed a peak for *BMAL1* between 03:00 and 11:00 hours, with a local peak at 11:00 hours on the second day of culture, like that observed in rat liver (96), which was delayed 4 hours to the lungs and adrenal glands of rats (97, 98), or 9 hours in the adrenal gland of monkeys (17). The antiphase of approximately 12 hours observed for *BMAL1* and *PER2* is similar to that of trophoblasts synchronized by serum shock (51). Alternatively, the circadian expression of the clock gene *PER1* is not detected during culture. Despite the above, we speculated that the expression of *PER1* must be high during the hours of the night and at the end of the day, different from what was reported in rat liver (96) or vascular smooth muscle cells (99), where maximum expression was observed during the night.

Our data suggest that the human placenta shows the same expression pattern as the peripheral oscillator-like lung (97) and a delay of 5–7 hours from the adrenal (17, 98). However, we observed an advanced phase of approximately 4 hours from the rat’s liver (96), and *PER2* expression is similar to that of the mouse placenta *ex vivo*. This pattern, after serum shock, is maintained in the culture of trophoblast cells (51). These data show the oscillation of the clock genes *BMAL1* and *PER2* in antiphase, which is related to the detected expression ratios of the *BMAL1/PER2* genes, and suggest that in the human placenta, there is an endogenous circadian clock with an autonomous capacity to work.

We showed the effect of melatonin on clock gene expression in the human placenta, similar to that reported over *BMAL1* and *PER2* in the pars tuberalis (100) and the adrenal gland (17, 101). Thus, our results suggest that melatonin has early effects on the expression of the clock genes as a chronobiotic agent, possibly via *BMAL1* inhibition and the posterior decrease of *PER1* and *PER2* expression. Another limitation of this approximation is that only a single concentration of melatonin (10 nM) was tested. Although this dose was selected based on its reported physiological relevance in placental and adrenal models, further dose–response studies will be necessary to fully establish the modulatory role of melatonin on placental circadian gene expression.

The circadian clock regulates the osteogenic potential by inhibiting BMAL1 expression (102), and the impaired expression of BMAL1 and PER1–2 causes tumor growth in mouse embryonic tissue (103). Alternatively, the knockdown of the clock gene *BMAL1* in carcinoma cells induces tumor growth when cells are injected subcutaneously, which may be mediated by the inhibition of apoptosis and reduction in the time that the cells remain in the G2/M phase (39). These antecedents suggest that the circadian system is closely related to the cell cycle in several peripheral tissues.

The *BMAL1/WEE1* ratio suggests that the circadian expression of *WEE1* increases at 03:00 hours, similar to a negative regulator of clock genes. The antiphase expression of *BMAL1* and *WEE1* detected here has been reported previously in the liver, with a peak for *WEE1* expression during the day/night transition and a peak for *BMAL1* during the night/day transition (96). Our results showed an antiphase of expression in the placenta for approximately 8 hours, suggesting the interplay of both clocks.

A limitation of our study is that clock gene expression was evaluated in whole placental explants rather than in isolated cells. While this approach allowed us to identify rhythmicity at the tissue level, it does not exclude the possibility that specific placental cell types, such as cytotrophoblasts or syncytiotrophoblasts, may exhibit distinct circadian dynamics. Future studies using isolated cell populations will be required to define cell type-specific rhythms and to clarify how melatonin modulates these cellular clocks.

**Figure 8:**
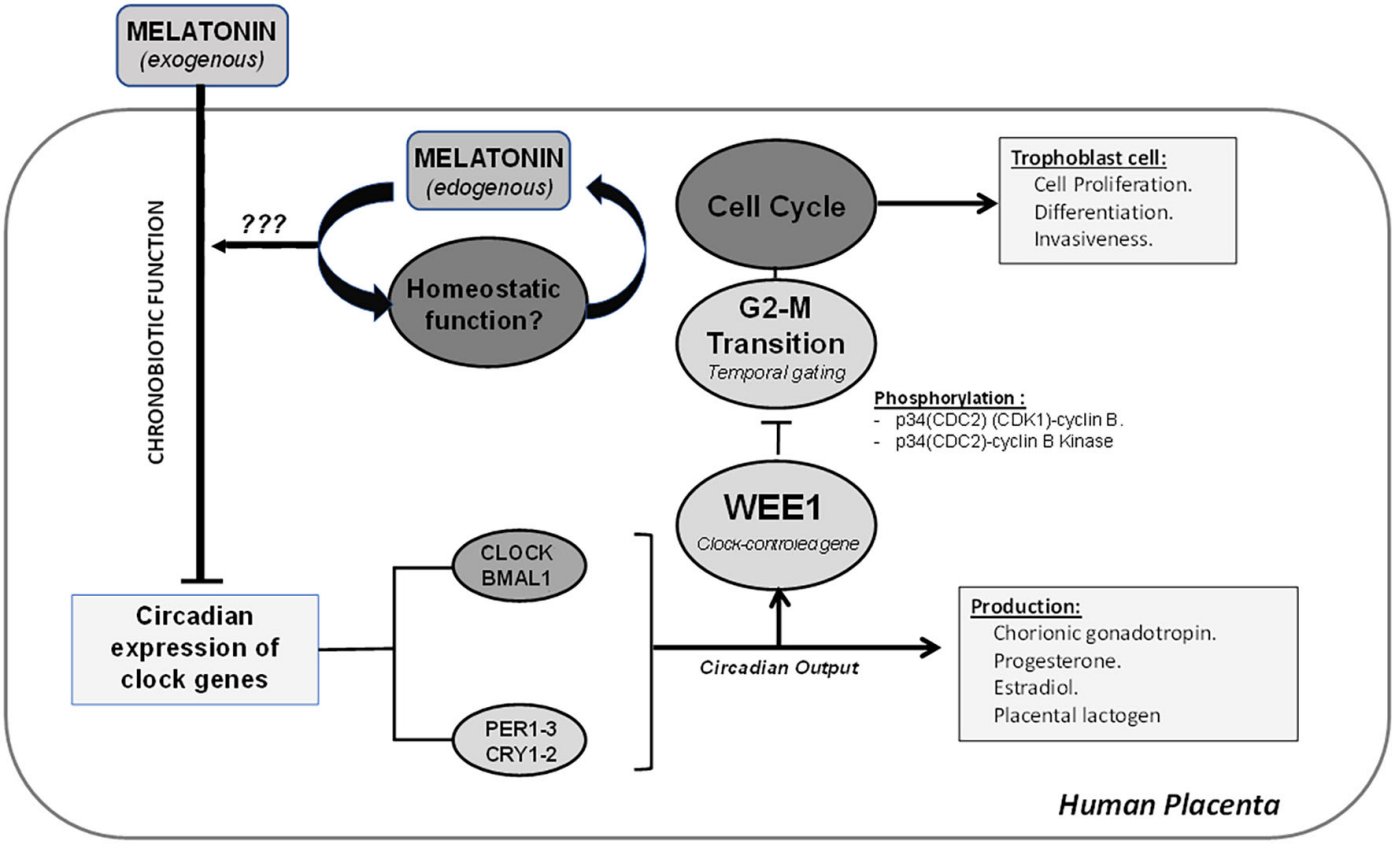
The placenta shows a circadian expression of clock genes, which can regulate the temporal gating of the cell cycle via modulation of mRNA expression of *WEE1* and inactivation of transition G2–M by phosphorylation of p34(CDC2) (CDK1)–cyclin B and p34(CDC2)–cyclin B kinase complex. The disruption of the circadian system of the placenta can modify critical processes, such as cell proliferation, differentiation, and invasiveness from trophoblast cells. Additionally, the placenta has the endogenous ability to produce melatonin, and it may play a homeostatic and antioxidant role in the placenta.

Our data suggest that variations in the expression levels of the clock genes *BMAL1*, *PER1-2*, and the cell cycle gene *WEE1* would correspond to a self-sustained placental capacity. The entry into mitosis by human placenta cells would be regulated by the clock genes, which would modulate *WEE1* expression levels by inhibiting the cell cycle. Furthermore, we showed an agonist role of melatonin in the cell cycle, decreasing the expression of clock genes *BMAL1*, *PER1*, and *PER2* and lowering the expression of *WEE1*. Similar to what was previously reported by Lanoix et al., we detected melatonin production in the human placenta (67). However, our results show that this production is sustained for at least 36 hours, suggesting a homeostatic role or protector against oxidative stress (31, 104) in the placenta that requires further investigation. These results support the existence of a circadian system–cell cycle interaction, modulated by the melatonin hormone. A graphical summary of the proposed mechanism is shown in **Figure 8**. This model may help explain how chronodisruption or the disruption of melatonin secretion could impact placental development and fetal health during pregnancy.

Our experimental limitation is that the explant culture-based approach assessed gene expression at the tissue level and did not directly measure functional cell cycle outcomes, such as those that occur with measurements of proliferation indices, BrdU incorporation, and flow cytometry. Future work should incorporate these readouts and include validation in human placental cell lines (e.g., BeWo and JEG-3) to define cell type-specific responses and determine whether melatonin suppresses clock gene expression at the cellular level. Another limitation is that we did not directly evaluate cell cycle progression or proliferation indices. This prevents us from linking the transcriptional changes of *WEE1* and other genes to functional outcomes. Future work should address this gap to strengthen the biological interpretation of our findings.

## Data Availability

The datasets presented in this study can be found in online repositories. The names of the repository/repositories and accession number(s) can be found in the article/supplementary material.

## References

[1] Matsuo T Yamaguchi S Mitsui S Emi A Shimoda F Okamura H. Control mechanism of the circadian clock for timing of cell division *in vivo* . Sci (New York N.Y.). (2003) 302:255–59. doi: 10.1126/science.1086271, PMID: 12934012

[2] Schibler U. Circadian rhythms. Liver regeneration clocks on. Sci (New York N.Y.). (2003) 302:234–35. doi: 10.1126/science.1090810, PMID: 14551421

[3] Gengatharan A Malvaut S Marymonchyk A. Adult neural stem cell activation in mice is regulated by the day/night cycle and intracellular calcium dynamics. Cell. (2021) 184:709–722.e13. doi: 10.1016/j.cell.2020.12.026, PMID: 33482084

[4] Lim GB. Surgery: circadian rhythms influence surgical outcomes. Nat Rev Cardiol. (2018) 15:5. doi: 10.1038/nrcardio.2017.186, PMID: 29143811

[5] Montaigne D Marechal X Modine T. Daytime variation of perioperative myocardial injury in cardiac surgery and its prevention by rev-erbα Antagonism: A single-centre propensity-matched cohort study and a randomised study. Lancet (London England). (2018) 391:59–69. doi: 10.1016/S0140-6736(17)32132-3, PMID: 29107324

[6] Kwon YS Jang SU Hwang SMI Tark H Kim JH Lee JJ. Effects of surgery start time on postoperative cortisol, inflammatory cytokines, and postoperative hospital day in hip surgery: randomized controlled trial. Medicine. (2019) 98:e158205. doi: 10.1097/MD.0000000000015820, PMID: 31192911 PMC6587638

[7] Ruby CL Major RJ Hinrichsen RD. “Regulation of tissue regeneration by the circadian clock. Eur J Neurosci. (2021) 53:3576–975. doi: 10.1111/ejn.15244, PMID: 33893679

[8] IARC Monographs Vol 124 group. Carcinogenicity of night shift work. Lancet Oncol. (2019) 20:1058–59. doi: 10.1016/S1470-2045(19)30455-3, PMID: 31281097

[9] Feillet C van der Horst GTJ Levi F Rand DA Delaunay F. Coupling between the circadian clock and cell cycle oscillators: implication for healthy cells and Malignant growth. Front Neurol. (2015) 6:96. doi: 10.3389/fneur.2015.00096, PMID: 26029155 PMC4426821

[10] Arafa K Emara M. Insights about circadian clock and molecular pathogenesis in gliomas. Front Oncol. (2020) 10:199. doi: 10.3389/fonc.2020.00199, PMID: 32195174 PMC7061216

[11] Crespo M Leiva M Sabio G. Circadian clock and liver cancer. Cancers. (2021) 13:36315. doi: 10.3390/cancers13143631, PMID: 34298842 PMC8306099

[12] Reid KJ Abbott. SM. “Jet lag and shift work disorder. Sleep Med Clinics. (2015) 10:523–355. doi: 10.1016/j.jsmc.2015.08.006, PMID: 26568127

[13] Valenzuela FJ Vera J Venegas C. Evidences of polymorphism associated with circadian system and risk of pathologies: A review of the literature. Int J Endocrinol. (2016) 2016:2746909. doi: 10.1155/2016/2746909, PMID: 27313610 PMC4893437

[14] Liang Y Wang S Huang X. Dysregulation of circadian clock genes as significant clinic factor in the tumorigenesis of hepatocellular carcinoma. Comput Math Methods Med. (2021) 2021:8238833. doi: 10.1155/2021/8238833, PMID: 34745328 PMC8570900

[15] Wendeu-Foyet M Cénée S Koudou Y. Circadian genes polymorphisms, night work and prostate cancer risk: findings from the EPICAP study. Int J Cancer. (2020) 147:3119–29. doi: 10.1002/ijc.33139, PMID: 32506468

[16] Bonmati-Carrion MA Arguelles-Prieto R Martinez-Madrid MJ. Protecting the melatonin rhythm through circadian healthy light exposure. Int J Mol Sci. (2014) 15:23448–500. doi: 10.3390/ijms151223448, PMID: 25526564 PMC4284776

[17] Valenzuela FJ Torres-Farfan C Richter HG. Clock gene expression in adult primate suprachiasmatic nuclei and adrenal: is the adrenal a peripheral clock responsive to melatonin? Endocrinology. (2008) 149:1454–61. doi: 10.1210/en.2007-1518, PMID: 18187542

[18] Lu Q Kim JY. Mammalian circadian networks mediated by the suprachiasmatic nucleus. FEBS J. (2021) 289(21):6589–604. doi: 10.1111/febs.16233, PMID: 34657394

[19] Finger A-M Kramer A. Peripheral clocks tick independently of their master. Genes Dev. (2021) 35:304–6. doi: 10.1101/gad.348305.121, PMID: 33649161 PMC7919411

[20] Sinturel F Gos P Petrenko V. Circadian hepatocyte clocks keep synchrony in the absence of a master pacemaker in the suprachiasmatic nucleus or other extrahepatic clocks. Genes Dev. (2021) 35:329–34. doi: 10.1101/gad.346460.120, PMID: 33602874 PMC7919413

[21] Finger A-M Kramer A. Mammalian circadian systems: organization and modern life challenges. Acta Physiol (Oxford England). (2021) 231:e135485. doi: 10.1111/apha.13548, PMID: 32846050

[22] Baydas G Gursu MF Cikim G. Effects of pinealectomy on the levels and the circadian rhythm of plasma homocysteine in rats. J Pineal Res. (2002) 33:151–55. doi: 10.1034/j.1600-079x.2002.02901.x, PMID: 12220329

[23] Cassone VM. The pineal gland influences rat circadian activity rhythms in constant light. J Biol Rhythms. (1992) 7:27–40. doi: 10.1177/074873049200700103, PMID: 1571591

[24] Farias TdSM de Oliveira AC Andreotti S. Pinealectomy interferes with the circadian clock genes expression in white adipose tissue. J Pineal Res. (2015) 58:251–61. doi: 10.1111/jpi.12211, PMID: 25626464

[25] Hartley S Dauvilliers Y Quera-Salva M-A. Circadian rhythm disturbances in the blind. Curr Neurol Neurosci Rep. (2018) 18:655. doi: 10.1007/s11910-018-0876-9, PMID: 30083814

[26] Liu J Clough SJ Hutchinson AJ Adamah-Biassi EB Popovska-Gorevski M Dubocovich ML. MT1 and MT2 melatonin receptors: A therapeutic perspective. Annu Rev Pharmacol Toxicol. (2016) 56:361–83. doi: 10.1146/annurev-pharmtox-010814-124742, PMID: 26514204 PMC5091650

[27] Matsumoto T Hess DL Kaushal KM Valenzuela GJ Yellon SM Ducsay CA. Circadian myometrial and endocrine rhythms in the pregnant rhesus macaque: effects of constant light and timed melatonin infusion. Am J Obstetrics Gynecol. (1991) 165:1777–84. doi: 10.1016/0002-9378(91)90032-m, PMID: 1750475

[28] Meyer-Bernstein EL Jetton AE Matsumoto SI Markuns JF Lehman MN Bittman EL. Effects of suprachiasmatic transplants on circadian rhythms of neuroendocrine function in golden hamsters. Endocrinology. (1999) 140:207–18. doi: 10.1210/endo.140.1.6428, PMID: 9886827

[29] Molinero P Soutto M Benot S Hmadcha A Guerrero JM. Melatonin is responsible for the nocturnal increase observed in serum and thymus of thymosin alpha1 and thymulin concentrations: observations in rats and humans. J Neuroimmunol. (2000) 103:180–88. doi: 10.1016/s0165-5728(99)00237-4, PMID: 10696913

[30] Paul MA Gray GW Lieberman HR. Phase advance with separate and combined melatonin and light treatment. Psychopharmacology. (2011) 214:515–23. doi: 10.1007/s00213-010-2059-5, PMID: 21069516

[31] Valenzuela FJ Vera J Venegas C Pino F Lagunas C. Circadian system and melatonin hormone: risk factors for complications during pregnancy. Obstetrics Gynecol Int. (2015) 2015:825802. doi: 10.1155/2015/825802, PMID: 25821470 PMC4363680

[32] Ko CH Takahashi JS. Molecular components of the mammalian circadian clock. Hum Mol Genet. (2006) 15:R271–277. doi: 10.1093/hmg/ddl207, PMID: 16987893

[33] Murakami H Nurse P. DNA replication and damage checkpoints and meiotic cell cycle controls in the fission and budding yeasts. Biochem J. (2000) 349:1–12. doi: 10.1042/bj3490001, PMID: 10861204 PMC1221113

[34] Weissbein U Benvenisty N Ben-David U. Quality control: genome maintenance in pluripotent stem cells. J Cell Biol. (2014) 204:153–635. doi: 10.1083/jcb.201310135, PMID: 24446481 PMC3897183

[35] Hunt T Sassone-Corsi P. Riding tandem: circadian clocks and the cell cycle. Cell. (2007) 129:461–645. doi: 10.1016/j.cell.2007.04.015, PMID: 17482541

[36] Gérard C Gonze D Goldbeter A. Effect of positive feedback loops on the robustness of oscillations in the network of cyclin-dependent kinases driving the mammalian cell cycle. FEBS J. (2012) 279:3411–315. doi: 10.1111/j.1742-4658.2012.08585.x, PMID: 22458764

[37] Masri S Cervantes M Sassone-Corsi P. The circadian clock and cell cycle: interconnected biological circuits. Curr Opin Cell Biol. (2013) 25:730–345. doi: 10.1016/j.ceb.2013.07.013, PMID: 23969329 PMC4573394

[38] Kelleher FC Rao A Maguire A. Circadian molecular clocks and cancer. Cancer Lett. (2014) 342:9–185. doi: 10.1016/j.canlet.2013.09.040, PMID: 24099911

[39] Zeng Z-L Wu M-W Sun J. Effects of the biological clock gene bmal1 on tumour growth and anti-cancer drug activity. J Biochem. (2010) 148:319–26. doi: 10.1093/jb/mvq069, PMID: 20576619

[40] Rotmensch S Celentano C Elliger N. Diurnal variation of human chorionic gonadotropin beta-core fragment concentrations in urine during second trimester of pregnancy. Clin Chem. (2001) 47:1715–17. doi: 10.1093/clinchem/47.9.1715, PMID: 11514413

[41] Díaz-Cueto L Barrios-de-Tomasi J Timossi C Méndez JP Ulloa-Aguirre A. More *In-Vitro* Bioactive, Shorter-Lived Human Chorionic Gonadotrophin Charge Isoforms Increase at the End of the First and during the Third Trimesters of Gestation. Mol Hum Reprod. (1996) 2:643–50. doi: 10.1093/molehr/2.9.643, PMID: 9239677

[42] Serón-Ferré M Ducsay CA Valenzuela GJ. Circadian rhythms during pregnancy. Endocrine Rev. (1993) 14:594–609. doi: 10.1210/edrv-14-5-594, PMID: 8262008

[43] Lee CK Moon DH Shin CS. Circadian expression of mel1a and PL-II genes in placenta: effects of melatonin on the PL-II gene expression in the rat placenta. Mol Cell Endocrinol. (2003) 200:57–66. doi: 10.1016/s0303-7207(02)00414-8, PMID: 12644299

[44] Doridot L Miralles F Barbaux S Vaiman D. Trophoblasts, invasion, and microRNA. Front Genet. (2013) 4:248. doi: 10.3389/fgene.2013.00248, PMID: 24312123 PMC3836020

[45] Frost JM Moore GE. The importance of imprinting in the human placenta. PloS Genet. (2010) 6:e10010155. doi: 10.1371/journal.pgen.1001015, PMID: 20617174 PMC2895656

[46] Gillespie AM Liyim D Goepel JR Coleman RE Hancock BW. Placental site trophoblastic tumour: A rare but potentially curable cancer. Br J Cancer. (2000) 82:1186–90. doi: 10.1054/bjoc.1999.1061, PMID: 10735504 PMC2363347

[47] Shoni M Nagymanyoki Z Vitonis AF. P-21-activated kinase-1, -4 and -6 and estrogen receptor expression pattern in normal placenta and gestational trophoblastic diseases. Gynecologic Oncol. (2013) 131:759–63. doi: 10.1016/j.ygyno.2013.09.010, PMID: 24051221

[48] Kobayashi Y Ye Z Hensch TK. Clock genes control cortical critical period timing. Neuron. (2015) 86:264–755. doi: 10.1016/j.neuron.2015.02.036, PMID: 25801703 PMC4392344

[49] Luiza JW Taylor SE Gao FF Edwards RP. Placental site trophoblastic tumor: immunohistochemistry algorithm key to diagnosis and review of literature. Gynecologic Oncol Case Rep. (2014) 7:13–5. doi: 10.1016/j.gynor.2013.11.001, PMID: 24624322 PMC3895280

[50] Frigato E Lunghi L Ferretti ME Biondi C Bertolucci C. Evidence for circadian rhythms in human trophoblast cell line that persist in hypoxia. Biochem Biophys Res Commun. (2009) 378:108–15. doi: 10.1016/j.bbrc.2008.11.006, PMID: 19000901

[51] Demarez C Assis LVMD Krohn M. The trophoblast clock controls transport across placenta in mice. Dev (Cambridge England). (2021) 148:dev197673. doi: 10.1242/dev.197673, PMID: 33913482

[52] Wharfe MD Mark PJ Waddell BJ. “Circadian variation in placental and hepatic clock genes in rat pregnancy. Endocrinology. (2011) 152:3552–605. doi: 10.1210/en.2011-0081, PMID: 21771885

[53] Akiyama S Ohta H Watanabe S. The uterus sustains stable biological clock during pregnancy. Tohoku J Exp Med. (2010) 221:287–98. doi: 10.1620/tjem.221.287, PMID: 20647694

[54] Pérez S Murias L Fernández-Plaza C. Evidence for clock genes circadian rhythms in human full-term placenta. Syst Biol Reprod Med. (2015) 61:360–66. doi: 10.3109/19396368.2015.1069420, PMID: 26247999

[55] Mark PJ Crew RC Wharfe MD Waddell BJ. Rhythmic three-part harmony: the complex interaction of maternal, placental and fetal circadian systems. J Biol Rhythms. (2017) 32:534–495. doi: 10.1177/0748730417728671, PMID: 28920512

[56] Tamura H Nakamura Y Pilar Terron M. Melatonin and pregnancy in the human. Reprod Toxicol (Elmsford N.Y.). (2008) 25:291–303. doi: 10.1016/j.reprotox.2008.03.005, PMID: 18485664

[57] Lanoix D Ouellette R Vaillancourt C. Expression of melatoninergic receptors in human placental choriocarcinoma cell lines. Hum Reprod (Oxford England). (2006) 21:1981–895. doi: 10.1093/humrep/del120, PMID: 16632463

[58] Soliman A Lacasse AA Lanoix D Sagrillo-Fagundes L Boulard V Vaillancourt C. Placental melatonin system is present throughout pregnancy and regulates villous trophoblast differentiation. J Pineal Res. (2015) 59:38–465. doi: 10.1111/jpi.12236, PMID: 25833399

[59] Lanoix D Beghdadi H Lafond J Vaillancourt C. Human placental trophoblasts synthesize melatonin and express its receptors. J Pineal Res. (2008) 45:50–605. doi: 10.1111/j.1600-079X.2008.00555.x, PMID: 18312298

[60] Clarkson-Townsend DA Bales KL Hermetz KE Burt AA Pardue MT Marsit CJ. Developmental chronodisruption alters placental signaling in mice. PloS One. (2021) 16:e02552965. doi: 10.1371/journal.pone.0255296, PMID: 34370755 PMC8351967

[61] Hong Y Won J Lee Y. Melatonin treatment induces interplay of apoptosis, autophagy, and senescence in human colorectal cancer cells. J Pineal Res. (2014) 56:264–74. doi: 10.1111/jpi.12119, PMID: 24484372

[62] Talib WH. Melatonin and cancer hallmarks. Molecules (Basel Switzerland). (2018) 23. doi: 10.3390/molecules23030518, PMID: 29495398 PMC6017729

[63] Su S-C Hsieh M-J Yang W-E Chung W-H Reiter RJ Yang S-F. Cancer metastasis: mechanisms of inhibition by melatonin. J Pineal Res. (2017) 62. doi: 10.1111/jpi.12370, PMID: 27706852

[64] Bejarano I Redondo PC Espino J. Melatonin induces mitochondrial-mediated apoptosis in human myeloid HL-60 cells. J Pineal Res. (2009) 46:392–400. doi: 10.1111/j.1600-079X.2009.00675.x, PMID: 19552762

[65] Hill SM Belancio VP Dauchy RT. Melatonin: an inhibitor of breast cancer. Endocrine-Rel Cancer Endocrine-Rel Cancer. (2015) 22:R183–204. doi: 10.1530/ERC-15-0030, PMID: 25876649 PMC4457700

[66] Blask DE Brainard GC Dauchy RT. Melatonin-depleted blood from premenopausal women exposed to light at night stimulates growth of human breast cancer xenografts in nude rats. Cancer Res. (2005) 65:11174–84. doi: 10.1158/0008-5472.CAN-05-1945, PMID: 16322268

[67] Lanoix D Guérin P Vaillancourt C. Placental melatonin production and melatonin receptor expression are altered in preeclampsia: new insights into the role of this hormone in pregnancy. J Pineal Res. (2012) 53:417–255. doi: 10.1111/j.1600-079X.2012.01012.x, PMID: 22686298

[68] Diallo A Coiffard B Desbriere R. Disruption of the expression of the placental clock and melatonin genes in preeclampsia. Int J Mol Sci. (2023) 24:2363. doi: 10.3390/ijms24032363, PMID: 36768691 PMC9917141

[69] Huang D Sherman BT Zheng X. Extracting biological meaning from large gene lists with DAVID. Curr Protoc Bioinf. (2009) 13. doi: 10.1002/0471250953.bi1311s27, PMID: 19728287

[70] Huang D Sherman BT Lempicki RA. “Systematic and integrative analysis of large gene lists using DAVID bioinformatics resources. Nat Protoc. (2009) 4:44–575. doi: 10.1038/nprot.2008.211, PMID: 19131956

[71] Cemerikic B Zamah R Ahmed MS. Opioid tolerance in human placenta due to *in vitro* methadone administration. J Pharmacol Exp Ther. (1995) 273:987–94. doi: 10.1016/S0022-3565(25)09689-2, PMID: 7791132

[72] Thordarson G Forsyth IA. Dopamine reduces the receptor binding activity and not the secretion rate of placental lactogen *in vitro* . J Reprod Fertil. (1984) 72:261–67. doi: 10.1530/jrf.0.0720261, PMID: 6512754

[73] Ilan J Pierce DR Hochberg AA Folman R Gyves MT. Increased rates of polypeptide chain elongation in placental explants from human diabetics. Proc Natl Acad Sci United States America. (1984) 81:1366–70. doi: 10.1073/pnas.81.5.1366, PMID: 6584885 PMC344834

[74] Nadri P Ansari-Mahyari S Jafarpour F. Melatonin accelerates the developmental competence and telomere elongation in ovine SCNT embryos. PloS One. (2022) 17:e0267598. doi: 10.1371/journal.pone.0267598, PMID: 35862346 PMC9302776

[75] Torres-Farfan C Richter HG Rojas-García P. Mt1 melatonin receptor in the primate adrenal gland: inhibition of adrenocorticotropin-stimulated cortisol production by melatonin. J Clin Endocrinol Metab. (2003) 88:450–58. doi: 10.1210/jc.2002-021048, PMID: 12519889

[76] Torres-Farfan C Richter HG Germain AM. Maternal melatonin selectively inhibits cortisol production in the primate fetal adrenal gland. J Physiol. (2004) 554:841–56. doi: 10.1113/jphysiol.2003.056465, PMID: 14673186 PMC1664788

[77] Torres-Farfan C Valenzuela FJ Mondaca M. Evidence of a role for melatonin in fetal sheep physiology: direct actions of melatonin on fetal cerebral artery, brown adipose tissue and adrenal gland. J Physiol. (2008) 586:4017–27. doi: 10.1113/jphysiol.2008.154351, PMID: 18599539 PMC2538916

[78] Martín M Macías M León J Escames G Khaldy H Acuña-Castroviejo Daríos. Melatonin increases the activity of the oxidative phosphorylation enzymes and the production of ATP in rat brain and liver mitochondria. Int J Biochem Cell Biol. (2002) 34:348–575. doi: 10.1016/s1357-2725(01)00138-8, PMID: 11854034

[79] Roth JA Rabin R Agnello K. Melatonin suppression of PC12 cell growth and death. Brain Res. (1997) 768:63–705. doi: 10.1016/S0006-8993(97)00549-0, PMID: 9369302

[80] Chomczynski P Nicoletta S. “Single-Step Method of RNA Isolation by Acid Guanidinium Thiocyanate-Phenol-Chloroform Extraction.” Analytical Biochemistry. (1987) 162 (1):156–59. doi: 10.1016/0003-2697(87)90021-2 2440339

[81] Filali S Bergamelli C Lamine Tall M. Formulation, stability testing, and analytical characterization of melatonin-based preparation for clinical trial. J Pharm Anal. (2017) 7:237–43. doi: 10.1016/j.jpha.2017.04.001, PMID: 29404044 PMC5790709

[82] Bates K Herzog ED. Maternal-fetal circadian communication during pregnancy. Front Endocrinol. (2020) 11:198. doi: 10.3389/fendo.2020.00198, PMID: 32351448 PMC7174624

[83] McCarthy R Jungheim ES Fay JC Bates K Herzog ED England SK. Riding the rhythm of melatonin through pregnancy to deliver on time. Front Endocrinol. (2019) 10:616. doi: 10.3389/fendo.2019.00616, PMID: 31572299 PMC6753220

[84] Galdames HA Torres-Farfan C Spichiger C. Impact of gestational chronodisruption on fetal cardiac genomics. J Mol Cell Cardiol. (2014) 66:1–11. doi: 10.1016/j.yjmcc.2013.10.020, PMID: 24200829

[85] Roman E Karlsson O. Increased Anxiety-like Behavior but No Cognitive Impairments in Adult Rats Exposed to Constant Light Conditions during Perinatal Development. Upsala J Med Sci. (2013) 118:222–75. doi: 10.3109/03009734.2013.821191, PMID: 23902426 PMC4190892

[86] Fontanetti PA Nervegna MT Vermouth NT Mandalunis PM. Prenatal exposure to continuous constant light alters endochondral ossification of the tibiae of rat pups. Cells Tis Org. (2014) 200:278–86. doi: 10.1159/000433520, PMID: 26278318

[87] Yajima M Matsumoto M Harada M Hara H Yajima T. Effects of Constant Light during Perinatal Periods on the Behavioral and Neuronal Development of Mice with or without Dietary Lutein. Biomed Res (Tokyo Japan). (2013) 34:197–2045. doi: 10.2220/biomedres.34.197, PMID: 23995056

[88] Olcese JM. Melatonin and female reproduction: an expanding universe. Front Endocrinol. (2020) 11:85. doi: 10.3389/fendo.2020.00085, PMID: 32210911 PMC7067698

[89] Dou Y Lin B Cheng H. The reduction of melatonin levels is associated with the development of preeclampsia: A meta-analysis. Hyperten Preg. (2019) 38:65–72. doi: 10.1080/10641955.2019.1581215, PMID: 30794002

[90] Warren WC Keisler DH Anthony RV. Synthesis and secretion of ovine placental lactogen and its biochemical properties. Domest Anim Endocrinol. (1990) 7:331–42. doi: 10.1016/0739-7240(90)90039-3, PMID: 1697232

[91] Downing GJ Maulik D Poisner AM. Human chorionic gonadotropin stimulates placental prorenin secretion: evidence for autocrine/paracrine regulation. J Clin Endocrinol Metab. (1996) 81:1027–30. doi: 10.1210/jcem.81.3.8772570, PMID: 8772570

[92] Rajashekhar G Loganath A Roy AC Wong YC. Expression and Localization of Angiogenin in Placenta: Enhanced Levels at Term over First Trimester Villi. Mol Reprod Dev. (2002) 62:159–66. doi: 10.1002/mrd.10116, PMID: 11984825

[93] Rubin LP Yeung B Vouros P Vilner LM Reddy GS. Evidence for human placental synthesis of 24,25-dihydroxyvitamin D3 and 23,25-dihydroxyvitamin D3. Pediatr Res. (1993) 34:98–104. doi: 10.1203/00006450-199307000-00023, PMID: 8356026

[94] Zheng J Li Y Weiss AR Bird IM Magness RR. Expression of endothelial and inducible nitric oxide synthases and nitric oxide production in ovine placental and uterine tissues during late pregnancy. Placenta. (2000) 21:516–24. doi: 10.1053/plac.1999.0504, PMID: 10940202

[95] Kudo Y Boyd CA. The role of L-tryptophan transport in L-tryptophan degradation by indoleamine 2,3-dioxygenase in human placental explants. J Physiol. (2001) 531:417–23. doi: 10.1111/j.1469-7793.2001.0417i.x, PMID: 11230514 PMC2278460

[96] Yamajuku D Inagaki T Haruma T. Real-time monitoring in three-dimensional hepatocytes reveals that insulin acts as a synchronizer for liver clock. Sci Rep. (2012) 2. doi: 10.1038/srep00439, PMID: 22666542 PMC3365280

[97] Hwang J-W Sundar IK Yao H Sellix MT Rahman I. Circadian clock function is disrupted by environmental tobacco/cigarette smoke, leading to lung inflammation and injury via a SIRT1-BMAL1 pathway. FASEB J: Off Publ Fed Am Soc Exp Biol. (2014) 28:176–945. doi: 10.1096/fj.13-232629, PMID: 24025728 PMC3868829

[98] Torres-Farfan C Mendez N Abarzua-Catalan L Vilches N Valenzuela GJ Seron-Ferre M. A circadian clock entrained by melatonin is ticking in the rat fetal adrenal. Endocrinology. (2011) 152:1891–900. doi: 10.1210/en.2010-1260, PMID: 21363938

[99] Chalmers JA Martino TA Tata N Ralph MR Sole MJ Belsham DD. Vascular circadian rhythms in a mouse vascular smooth muscle cell line (Movas-1). Am J Physiol Regul Integr Comp Physiol. (2008) 295:R1529–1538. doi: 10.1152/ajpregu.90572.2008, PMID: 18768761

[100] Dardente H Menet JérômeS Poirel VJ. Melatonin induces cry1 expression in the pars tuberalis of the rat. Brain Res Mol Brain Res. (2003) 114:101–6. doi: 10.1016/S0169-328X(03)00134-7, PMID: 12829319

[101] Campino C Valenzuela FJ Torres-Farfan C. Melatonin exerts direct inhibitory actions on ACTH responses in the human adrenal gland. Hormone Metab Res = Horm Und Stoffwechselforschung = Horm Et Métaboli. (2011) 43:337–42. doi: 10.1055/s-0031-1271693, PMID: 21332028

[102] Gambardella A Nagaraju CK O’Shea PJ. Glycogen synthase kinase-3α/β Inhibition promotes *in vivo* amplification of endogenous mesenchymal progenitors with osteogenic and adipogenic potential and their differentiation to the osteogenic lineage. J Bone Min Res: Off J Am Soc Bone Min Res. (2011) 26:811–21. doi: 10.1002/jbmr.266, PMID: 20939016

[103] Lee J Kim M-S Li R. Loss of bmal1 leads to uncoupling and impaired glucose-stimulated insulin secretion in β-cells. Islets. (2011) 3:381–88. doi: 10.4161/isl.3.6.18157, PMID: 22045262 PMC3329519

[104] Richter HG Hansell JA Raut S Giussani DA. Melatonin improves placental efficiency and birth weight and increases the placental expression of antioxidant enzymes in undernourished pregnancy. J Pineal Res. (2009) 46:357–645. doi: 10.1111/j.1600-079X.2009.00671.x, PMID: 19552758

